# Robotic-assisted compartmental dissection for retroperitoneal sarcoma: a feasibility study on cadavers with the da Vinci Xi system

**DOI:** 10.1007/s11701-026-03869-6

**Published:** 2026-08-29

**Authors:** Jonas Wakker, Adrian Ohlde, Victoria van Rüth, Simone Schewe, Nathaniel Melling, Felix Nickel, Oliver Mann, Thilo Hackert, Volker Spindler, Sophie Kurmann, Rouven Berndt, Markus Albertsmeier, Jens Jakob, Anna Duprée

**Affiliations:** 1 Department of General, Visceral and Thoracic Surgery, University Medical Center Hamburg-Eppendorf, Martinistraße 52, 20246 Hamburg, Germany; 2 Institute of Anatomy and Experimental Morphology, University Medical Center Hamburg- Eppendorf, Martinistraße 52, 20246 Hamburg, Germany; 3 University Heart & Vascular Center Hamburg Clinic of Vascular Medicine, University Medical Center Hamburg- Eppendorf, Martinistraße 52, 20246 Hamburg, Germany; 4 Department of General, Visceral and Transplantation Surgery, Ludwig- Maximilians-Universität (LMU) Munich, LMU University Hospital, Munich, Germany; 5 Sarcoma Unit, Department of Surgery, University Medical Center and Medical Faculty Mannheim, Heidelberg University, Mannheim, Germany

**Keywords:** Retroperitoneal sarcoma, Robotic surgery, Da Vinci Xi, Compartmental resection, Cadaveric feasibility study, IDEAL framework

## Abstract

Retroperitoneal sarcomas (RPS) pose major technical challenges because of their size, anatomical complexity, and need for multivisceral resection. We evaluated the feasibility of a standardized robotic approach for right- and left-sided retroperitoneal sarcoma (RPS) compartmental resection using the da Vinci Xi system in tumour-free human cadavers. In a two-phase IDEAL-D Stage 0 cadaveric study, two sarcoma surgeons first compared four trocar configurations in two cadavers to identify optimal port templates (Phase 1). Complete six-stage compartmental resection following a standardised protocol was then performed once on each side in a third cadaver (Phase 2). The confirmatory dissection was assessed with a 5-point anatomical reach rating per predefined landmark, an adapted System Usability Scale (SUS), and a structured safety surrogate log. Phase 1 yielded a horizontal suprasymphysial port template for the right side and an oblique xiphoid-to-right-lower-quadrant template for the left. In Phase 2, all six stages were completed bilaterally; all predefined landmarks were reached (pooled median reach 5/5, range 3–5; inter-rater agreement 90%). Three-dimensional visualization and instrument articulation were rated highly, the platform scored 72.5/100 on the System Usability Scale, and no unintended major injury occurred. A robotic platform can reproduce the anatomical exposure required for the six-stage compartmental resection concept in a tumour-free cadaveric model, using side-specific port templates. Because no tumour was present, these findings address anatomical access rather than oncological resection. Investigation in tumour-bearing models is required before clinical evaluation, which must remain restricted to highly selected patients at expert centres.

**Supplementary Information:**

The online version contains supplementary material available at https://doi.org/10.1007/s11701-026-03869-6.

## Introduction

Retroperitoneal sarcomas (RPS) pose substantial surgical challenges due to their large size, heterogeneous histology, proximity to major vascular and visceral structures, and high risk of local recurrence. Radical multivisceral resection via open surgery is the standard approach to achieve an oncologically adequate resection [1], but it entails considerable morbidity and still carries a high risk of local recurrence [2]. Minimally invasive and robotic techniques, particularly the da Vinci surgical system, have demonstrated benefits across various tumour types — especially in colorectal and upper gastrointestinal surgery — including reduced perioperative trauma, shorter hospitalization, and faster recovery.

Despite these advantages, robotic-assisted surgery in sarcoma remains limited. The size of retroperitoneal sarcomas, combined with the multivisceral nature of the resection and the need for a large incision to extract the specimen, appears to contradict a minimally invasive surgery (MIS) approach.

“Just because we can does not mean we should” was the conclusion drawn in 2018 by Gronchi and colleagues in response to a National Cancer Database analysis of MIS in RPS by Gani et al. [3, 4]. Where, then, do we stand seven years later?

Across modern multi-institutional series, 5-year overall survival for primary RPS ranges from approximately 40% to 70%, and 5-year local recurrence rates from 25% to 50% — both heavily dependent on histologic subtype and grade. For well-differentiated liposarcoma (G1), 5-year overall survival approaches 85–90%, but local recurrence approximates 40% at 5 years and may exceed 50% with longer follow-up. For high-grade (G2/G3) dedifferentiated liposarcoma 5-year overall survival drops to approximately 50–60%, with comparable 5-year local recurrence rates of 40–50% despite established multivisceral compartment resection [5, 6]. Improvements to the current standard of care are therefore urgently needed; however, the median tumour size of approximately 20 cm in primary RPS presents considerable challenges for minimally invasive approaches [5]. The rationale for the present study was not solely to reduce incision size, but primarily to explore further potential advantages of robotic-assisted surgery — particularly the ability to operate beneath the tumour while maintaining high-resolution visualization and enabling precise dissection of structures such as the femoral nerve and iliac vessels while preserving an adequate resection margin.

Given the increasing adoption of robotic-assisted surgery for many solid tumours, including prostate, colorectal, and upper gastrointestinal malignancies [7, 8], it is appropriate to explore its potential application in sarcoma surgery. Standardized protocols for feasibility, port placement, and anatomical reach are, however, still lacking. To address this gap, we conducted a two-phase cadaveric feasibility study using the da Vinci surgical system to establish a structured robotic approach for RPS surgery. The cadavers used did not contain tumours, but they allowed systematic evaluation of the critical anatomy relevant to compartmental resection. This proof-of-concept study provides preliminary data on technical feasibility, port placement, and operative workflow. Its scope is deliberately confined to anatomical access and landmark accessibility in the tumour-free retroperitoneum, as the first preclinical step of a stepwise IDEAL-aligned development pathway.

## Methods

### Study design and reporting framework

This was an experimental cadaveric study evaluating the feasibility and technical performance of da Vinci Xi-assisted multivisceral compartmental dissection for simulated right- and left-sided RPS, focusing on anatomical reach, instrument mobility, and the definition of side-specific port placement patterns. The study corresponds to IDEAL-D Stage 0 (preclinical device evaluation) within the IDEAL framework [9, 10]. Reporting follows the CACTUS guidelines for cadaveric training and surgical studies [11], with methodological quality self-appraised against the QUACS scale [12]. It was conducted in a dedicated experimental operating room using a da Vinci Xi^®^ Surgical System (Intuitive Surgical Inc., Sunnyvale, CA, USA) under routine operating standards, in collaboration with the Institute of Anatomy and Experimental Morphology, University Medical Center Hamburg-Eppendorf.

Figures were assembled using GIMP 2.10.

### Cadaver models and study phases

Three human cadavers with intact abdominal anatomy and no known intra-abdominal malignancy or prior major abdominal surgery were used. Cadaver suitability was assessed using a five-item Likert questionnaire (1–10 scale), orientated on the McMaster Embalming Scale [13]. The study proceeded in two sequential phases (Supplementary Table S1). Cadavers No. 1 (fresh-frozen) and No. 2 (Fix for Life [F4L] immersion, used immediately after fixation) were used for iterative comparison of trocar configurations (Phase 1); Cadaver No. 3 (F4L immersion, stored three months after fixation) was used for the confirmatory six-stage compartmental resection on each side (Phase 2). This design allowed subjective comparison of tissue handling across preservation techniques.

No tumour was present in any cadaver, and no physical tumour surrogate — phantom, balloon, packing, or other space-occupying device — was used. The conceptual tumour centre referred to below served solely to standardize port planning relative to the anatomy of the two reference clinical cases.

### Reference clinical cases

Two recent clinical cases from our institution served as the anatomical reference for compartmental dissection on each side. For the right-sided compartment, the reference was a 70-year-old patient with a cT4 high-grade dedifferentiated liposarcoma — the first recurrence after external R1 resection two years earlier; the tumour infiltrated the right kidney, did not extend cranially beyond the hepatic flexure, and did not extend caudally beyond the origin of the internal iliac vessels. For the left-sided compartment, the reference was a 68-year-old patient with a cT4 G2 dedifferentiated liposarcoma ventral to the left kidney, without involvement of pancreas or spleen, and with caudal extent limited to the pelvic inlet.

### Phase 1 — Trocar configuration optimization

Four trocar configurations were trialled iteratively for right- and left-sided access across Cadavers No. 1 and No. 2: an oblique line from xiphoid to right lower quadrant, an oblique line from xiphoid to left lower quadrant, a vertical paramedian arrangement, and a horizontal suprasymphysial line. In each, the camera port was placed approximately 20 cm from the conceptual tumour centre, with the remaining trocars in line and approximately 6–8 cm apart. Dissections in this phase were exploratory and partial: their purpose was to compare port arrangements, not to complete the six-stage protocols. Both surgeons jointly assessed ergonomics, arm conflict, external collision, and qualitative reach toward the principal anatomical targets, and selected the final port templates by consensus. Instrument choice (monopolar scissors, fenestrated bipolar, Tip-Up Fenestrated Grasper, Vessel sealer, Maryland bipolar) and camera position were adjusted as needed.

### Phase 2 — Confirmatory six-stage dissection

Using the port templates selected in Phase 1, the six-stage compartmental resection protocols of Radaelli et al. (right side) [14] and Baia et al. (left side) [15] were executed once on each side in Cadaver No. 3, conceptually dividing the abdomen into right and left retroperitoneal multivisceral compartments per current international consensus [1]. The six key stages on each side are summarized in Table 1; the full landmark inventory is provided in Supplementary Tables S2 and S3. Reconstruction steps (ileocolic and colorectal anastomoses) were conceptually planned but not fashioned, since the focus was on exposure and compartmental dissection rather than anastomotic technique.

**Table 1:** Six-stage standardized protocols for right- and left-sided retroperitoneal compartmental resection (summary)

Stage	Right-sided protocol Radaelli et al. [14]	Left-sided protocol Baia et al. [15]
**1**	Access and exploration (originally via midline laparotomy)	Access and exploration (originally via midline laparotomy); identification of IMV at the duodenojejunal junction
**2**	Gastrocolic ligament division; transverse colon mobilization; ileocolic vessel ligation; central SMV/SMA exposure	Division of transverse colon; assessment of pancreas and spleen; division of gastrocolic ligament; IMV ligation
**3**	Kocher manoeuvre with duodenal mobilization; exposure of pancreatic head; posterior dissection toward vena cava	Medialization of spleen and pancreas; division of splenorenal ligament; release of diaphragmatic attachments; exposure of left retroperitoneum
**4**	Dissection along the inferior vena cava; ligation of right gonadal, renal artery, and renal vein	Aortic dissection; ligation of left renal artery and vein and of the IMA
**5**	Lateral peritonectomy; iliac vessel exposure; ureter identification; preservation of obturator and femoral nerves; partial distal psoas division	Posterolateral peritonectomy; division of rectum and distal psoas; peritonectomy to costodiaphragmatic fold; ligation of gonadal vessels and left ureter; iliac vessel dissection; femoral nerve isolation
**6**	Dissection along the spine and retrocaval region; partial psoas resection; right adrenalectomy; mobilization of right diaphragmatic fold and crus	Dissection of the psoas muscle; liberation of the costodiaphragmatic fold

IMA inferior mesenteric artery, IMV inferior mesenteric vein, SMA superior mesenteric artery, SMV superior mesenteric vein

### Assessment framework

A structured assessment framework was prespecified and is fully described in the Supplementary Methods. Three instruments were applied to the Phase 2 confirmatory dissection: (i) a novel pragmatic 5-point Likert anatomical reach rating per predefined landmark (1 = no useful reach; 5 = full unrestricted reach), with limiting-factor codes (R = reach; A = arm conflict; V = visualization; T = tissue limitation; O = other) recorded for any landmark scoring below 5; (ii) an adapted System Usability Scale (SUS) [16, 17]; and (iii) a structured safety surrogate log for predefined critical structures (aorta, vena cava, ureters, main renal vessels, femoral and obturator nerves, duodenum, pancreas, spleen, bowel). Cadaver tissue handling was rated for all three cadavers using the five-item Likert questionnaire described above (see Supplementary Methods). Photographic documentation of key steps was obtained for all phases.

Validated robotic skill instruments (Global Evaluative Assessment of Robotic Skills, GEARS; Cumulative Assessment of Robotic Skills, CARS) were deliberately not applied: they assess operator dexterity and console performance, whereas the prespecified endpoints here were anatomical access and the comparison of port configurations. Skill-based scoring instruments are planned for the subsequent tumour-model and clinical phases, where operator performance and learning curves become relevant outcomes. The SUS was completed once as a joint consensus rating rather than individually, because both surgeons worked as a single console team and the instrument served to characterize the platform in this task context rather than to compare users; this deviation is addressed under Limitations.

### Statistical analysis

All measures are reported descriptively. For the anatomical reach rating, per-stage and overall median (range) scores are reported separately for each side, pooled across the two raters. Inter-rater agreement is reported as the proportion of exact agreement and as Cohen’s κ — unweighted, and with linear and quadratic distance weights — per side and pooled, with 95% confidence intervals obtained by non-parametric bootstrap. Given the small confirmatory N, no inferential testing is performed; the framework is intended for descriptive proof-of-concept reporting consistent with IDEAL-D Stage 0.

### Ethics

All procedures complied with institutional body-donation and cadaveric-research regulations of the Institute of Anatomy and Experimental Morphology, University Medical Center Hamburg-Eppendorf, and followed prevailing ethical standards for the use of human bodies in surgical education and research.

Cadavers were registered in the body donation program of the Institute of Anatomy and Experimental Morphology, University Medical Center Hamburg-Eppendorf. Written informed consent was obtained from both donors and next of kin prior to decease for the purpose of teaching and research.

## Results

### Phase 1 — Selection of side-specific port templates

**Fig. 1:**
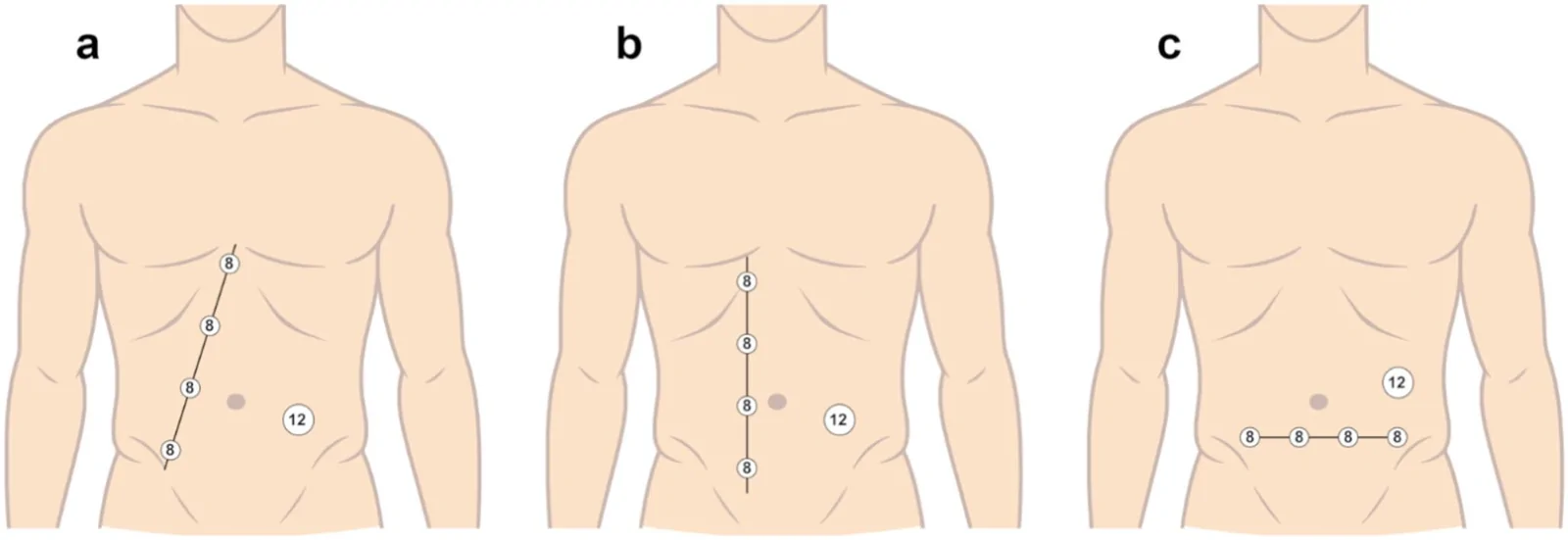
Final trocar positioning. **a** Port template for left-sided RPS. **b** Adjusted port template for RPS located in the left upper quadrant. **c** Port template for right-sided RPS

Across Cadavers No. 1 and No. 2, four trocar configurations were trialled. For right-sided access, the selected template was a horizontal suprasymphysial linear arrangement analogous to that used in oncologic right hemicolectomy with complete mesocolic excision — essentially an extension of the classic Pfannenstiel incision (Fig. 1c). Both surgeons judged this arrangement to provide stable triangulation toward the ileocolic root and central mesenteric vessels, an unobstructed line of sight toward the duodenum, pancreatic head, renal hilum and vena cava, and sufficient caudal reach for iliac and femoral nerve dissection without excessive external arm collision. Alternative placements (more cranial, vertical, or oblique arrays) were associated either with increased arm conflict when transitioning between central and pelvic targets or with reduced ability to work comfortably along the central vessels.

An exception to the right-sided template is provided by RPS with extension into the right inguinal canal or pelvis; depending on cranio-caudal diameter, an open approach may be necessary to achieve adequate exposure. However, for tumours in the right lower abdomen, right groin, or small pelvis without cranial extension to the liver or diaphragm, an oblique trocar arrangement provided good exposure in Phase 1, with the first trocar placed immediately right latero-caudal to the xiphoid and the line extending medially and downward to the left anterior superior iliac spine. Intraoperative re-adjustment of the targeted anatomy — standard practice in colorectal robotic surgery — proved helpful during the prolonged dissection.

For left-sided access, the selected template was an oblique line from the xiphoid to the right lower quadrant (Fig. 1a). This configuration facilitated wide-angle visualization of the left upper quadrant, including the spleen, pancreas, and splenic flexure; direct access to the aorta, left renal vessels, and IMA; and a smooth transition to pelvic, iliac, and rectal dissection. It also permits a safe transversorectostomy, analogous to established procedures in colorectal surgery. More lateral or horizontal arrays limited medial reach to the aorta and made deep pelvic work cumbersome. For left-sided sarcomas predominantly located in the left upper abdominal quadrant, the trocar template can be aligned vertically and paramedially to the right to improve access to the costodiaphragmatic fold, spleen, and pancreas (Fig. 1b).

### Phase 2 — Confirmatory six-stage dissection

Using the templates selected in Phase 1, the six-stage compartmental resection was performed on both the right and the left side. All predefined anatomical landmarks were reached on both sides. No step was abandoned because of inadequate reach or insurmountable loss of exposure.

**Fig. 2:**
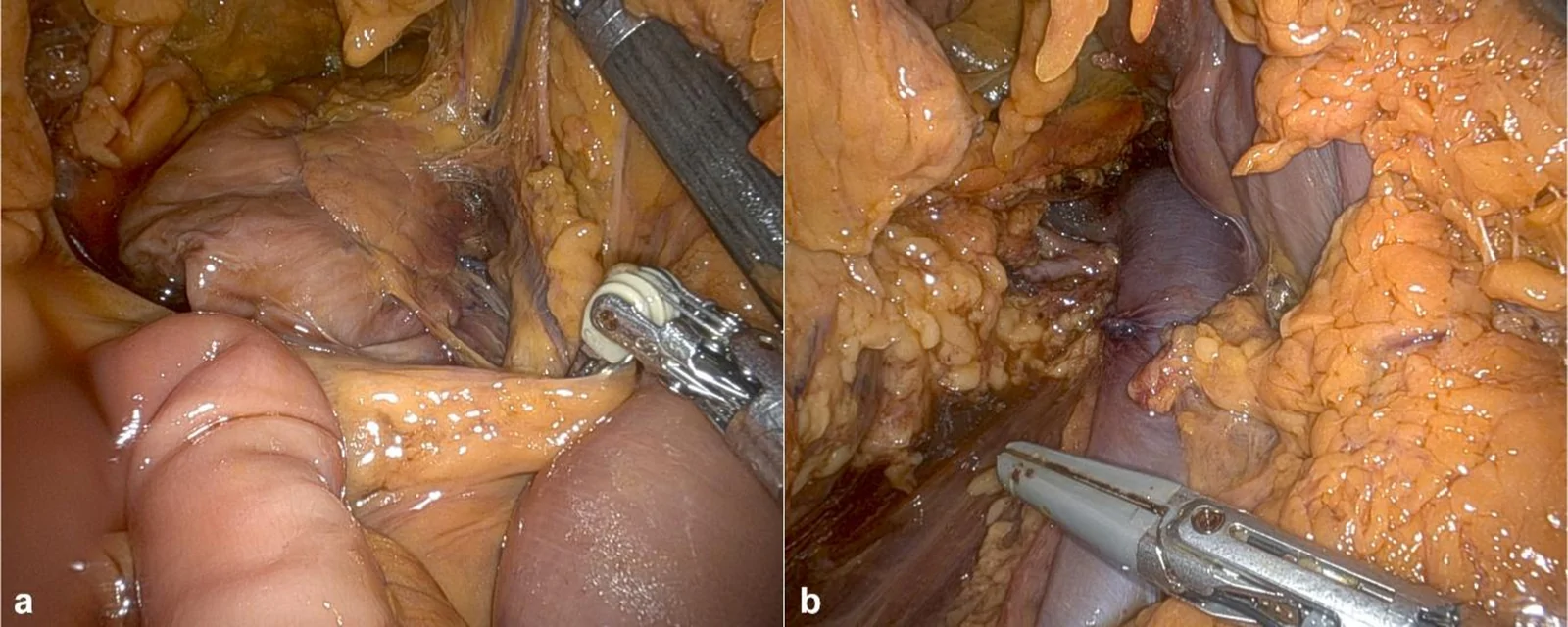
Right-sided dissection. **a** Exposure of the pancreatic head, duodenum, middle colic vessels and ileocolic vein. **b** Exposure of the inferior vena cava, right renal pedicle, right quadratus lumborum and psoas muscle

On the right side, the configuration provided stable exposure of the right colon, duodenum, pancreatic head, inferior vena cava, right renal hilum, and iliac vessels, with caudal access toward the psoas muscle, ureter, and femoral nerve (Fig. 2a, b). Posterior dissection along the spine, retroperitoneal fat clearance including a layer of psoas up to the right diaphragmatic crus, and the retrohepatic space were feasible, confirming adequate cranial and dorsal reach.

**Fig. 3:**
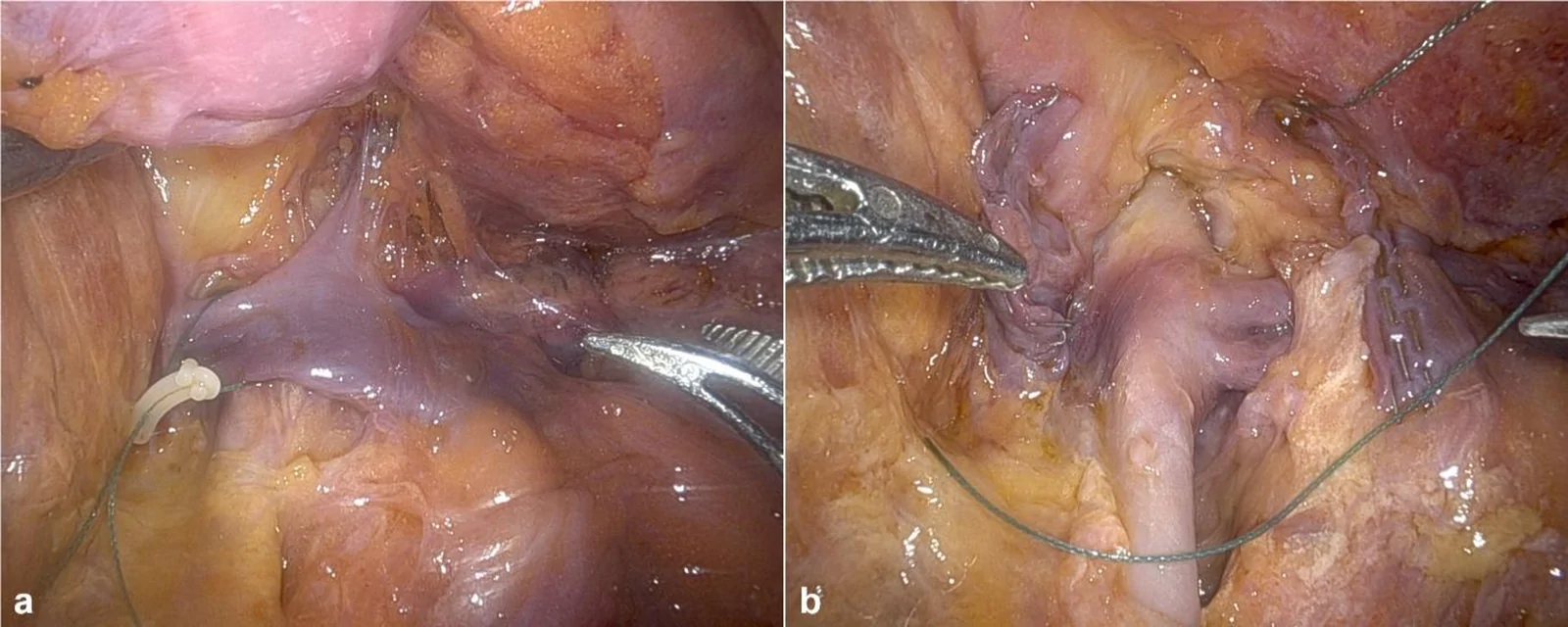
Left-sided dissection. **a** Exposure of the left renal vein and the aorta. **b** After division of the left renal vein the aorta and two renal arteries are exposed

**Fig. 4:**
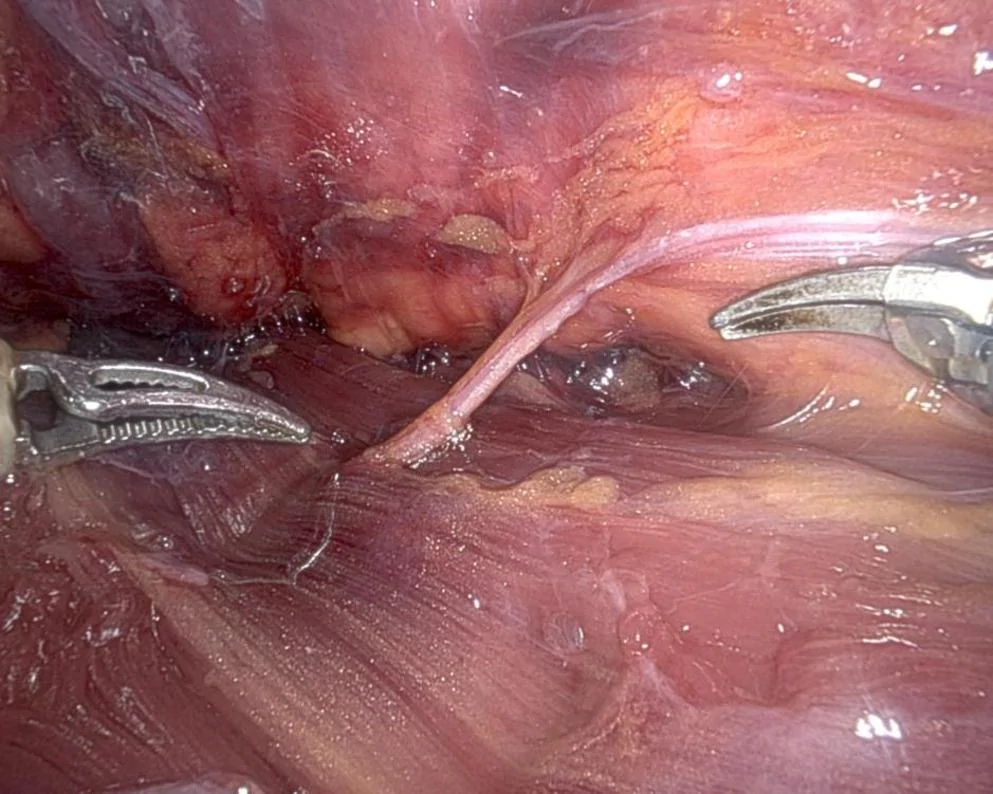
Left-sided dissection. Visualization of the left retroperitoneal space and dissection of the parietal nerves of the lumbar plexus

On the left side, the IMV was consistently identified at the duodenojejunal junction. Division of the transverse colon with opening of the gastrocolic ligament enabled medial-to-lateral mobilization, allowing access to and visualization of the central vascular axis, including the aorta and left renal pedicle (Fig. 3a, b). Mobilization of the pancreatic body and tail with the spleen, achieved by division of the splenorenal ligament and diaphragmatic attachments, gave excellent exposure of the left retroperitoneum; consequently, a distal splenopancreatectomy was feasible. Safe dissection of the aorta with ligation of the left renal vessels and IMA was accomplished while accommodating cranial dissection toward the costodiaphragmatic recess. Posterolateral dissection allowed clear visualization of the left retroperitoneal space, including the parietal nerves of the lumbar plexus in their retroperitoneal course (Fig. 4). In contrast to the right side, repositioning of the target anatomy with redocking was required for optimal instrument alignment — first targeting the splenic flexure, then the upper rectum after completion of central and upper-quadrant dissection.

### Anatomical reach rating

Both surgeons independently rated all 50 predefined landmarks of the six-stage protocols (26 right-sided, 24 left-sided). Scores of 4 (good reach) or 5 (full reach) were assigned for the vast majority, with no landmark scoring 1 (no useful reach) on either side; the pooled median was 5 (range 3–5) on both sides (Table 2). Scores of 3 (reach with adjustment) were confined to access and exploration after docking (Stage 1, both sides) and division of the rectum on the left (Stage 5), which required redocking from the splenic flexure onto the sigmoid. The dominant limiting factors were arm conflict during pelvic dissection and reach limitation during left-sided medialization of the pancreas and spleen.

Inter-rater exact agreement was 92.3% (right), 87.5% (left), and 90.0% (45/50) overall. Four of the five disagreements were single-point; the only two-point disagreement concerned the Stage 1 access-and-exploration landmark (Surgeon 1 = 3, Surgeon 2 = 5 on each side), reflecting differing interpretations of post-docking circumferential visibility. Quadratic-weighted Cohen’s κ was 0.66 (right), 0.63 (left), and 0.64 pooled — substantial agreement by the Landis and Koch benchmarks [18] — though the bootstrap 95% confidence intervals were wide (pooled 0.38–0.93), as expected for a single confirmatory dissection per side. Item-level scores, limiting-factor codes, and the full agreement statistics are provided in Supplementary Tables S2 and S3 and Supplementary Methods Sect. 3.4.

**Table 2:** Anatomical reach rating summary across the six stages of the confirmatory dissection (Cadaver No. 3). Each landmark was rated independently by both surgeons; scores (5-point Likert: 1 = no useful reach, 5 = full unrestricted reach) and medians are pooled across the two raters

Side	Stage	Landmarks (*n*)	Pooled median (range)	Ratings (*N*)	Scores 5 / 4 / 3	Main limiting factor
Right	1 — Access and exploration	1	4 (3–5)	2	1 / 0 / 1	Limited circumferential visibility after docking
	2 — Colon and central vessels	5	5 (4–5)	10	9 / 1 / 0	Arm conflict toward SMV/SMA
	3 — Kocher / pancreatic head	4	5 (4–5)	8	6 / 2 / 0	Arm conflict toward vena cava
	4 — IVC and right renal pedicle	4	5	8	8 / 0 / 0	—
	5 — Lateral peritonectomy and pelvis	7	4	14	0 / 14 / 0	Steep instrument angle into pelvis
	6 — Spine, retrocaval region and diaphragm	5	5 (4–5)	10	8 / 2 / 0	Reach to diaphragmatic crus
Right — overall	*Exact agreement 92.3%; quadratic-weighted κ 0.66*	**26**	**5 (3–5)**	**52**	**32 / 19 / 1**	—
Left	1 — Access and IMV identification	2	5 (3–5)	4	3 / 0 / 1	Limited circumferential visibility after docking
	2 — Colon, pancreas/spleen, IMV	4	5	8	8 / 0 / 0	—
	3 — Medialization / left retroperitoneum	5	4 (4–5)	10	2 / 8 / 0	Reach to pancreatic body/tail and diaphragm
	4 — Aorta and left renal pedicle	4	5	8	8 / 0 / 0	—
	5 — Posterolateral peritonectomy and pelvis	7	4.5 (3–5)	14	7 / 6 / 1	Redocking onto sigmoid; arm conflict toward iliac vessels and nerve
	6 — Psoas and costodiaphragmatic fold	2	4.5 (4–5)	4	2 / 2 / 0	Reach to costodiaphragmatic fold
Left — overall	*Exact agreement 87.5%; quadratic-weighted κ 0.63*	**24**	**5 (3–5)**	**48**	**30 / 16 / 2**	—

Each of the 50 landmarks was rated by both surgeons; “Ratings (N)” is therefore twice the number of landmarks. Pooled median, range, and the 5/4/3 score distribution are computed over both raters’ scores. Inter-rater agreement statistics (per side and pooled, including unweighted and linear-weighted κ with bootstrap 95% confidence intervals) are detailed in Supplementary Methods Sect. 3.4. IVC inferior vena cava, IMV inferior mesenteric vein, SMA superior mesenteric artery, SMV superior mesenteric vein

### Instrument mobility, visualization, and system usability

Three-dimensional visualization, instrument articulation, and the steadiness of the robotic platform were judged particularly advantageous during dissection around the vena cava, renal hilum, and iliac vessels, and during dissection of the femoral nerve. The retroperitoneal view and clearance, including the ability to work beneath the conceptual tumour position while maintaining high-resolution visualization, was rated as a major qualitative advantage over open practice. Minor arm conflicts during transitions toward the pelvis were mitigated by small adjustments of inter-arm distance and camera angulation. Performing certain steps laparoscopically (e.g., positioning the small intestine before robotic docking) was advantageous and allowed selected steps to be carried out against the robot’s primary orientation.

On the adapted System Usability Scale (SUS), completed once as a joint consensus rating by both surgeons after the Phase 2 confirmatory dissection, the score was 72.5/100 — above the established average benchmark of 68 and within the acceptable range per Bangor et al. [17]. Item-level responses rated integration of functions (item 5 = 5 of 5) and ease of use (item 3 = 4 of 5) highly, whereas frequency of intended use (item 1 = 3 of 5) and confidence in use (item 9 = 3 of 5) were more reserved, consistent with the early-adoption context of an IDEAL-D Stage 0 study. Within the cadaveric setting, the surgeons judged that the robotic approach allowed uncompromised completion of the six-stage protocols when assessed purely on anatomical reach and dissection capability.

### Safety surrogates

No unintended major damage to critical structures was observed during the Phase 2 confirmatory dissections. The structured safety log (Supplementary Methods) audited 16 critical anatomical sites: contact during dissection was recorded for seven sites (aorta, inferior vena cava, right renal artery/vein, inferior mesenteric vein, left ureter, duodenum/pancreatic head, bowel), as expected. Visible minor changes were noted at only two sites — inferior vena cava and bowel — both grasper impressions without transection or perforation. No major technical limitations were encountered after optimization of port placement.

### Cadaver preservation and tissue handling

All three cadavers provided good colour and anatomical quality. The F4L-immersion cadaver stored three months after fixation (Cadaver No. 3) was rated by both surgeons as providing the most realistic tactile feedback and tissue pliability. The fresh-frozen cadaver (No. 1) showed slightly restricted tissue mobility; reduced mesenteric elasticity limited the range of motion during medialization, though this progressively diminished. The F4L cadaver used immediately after fixation (No. 2) tended to ooze, requiring near-continuous suctioning, but the tissue itself was very realistic during dissection. Embryonic boundary layers were clearly identifiable in all cadavers, and lateral peritonectomy was easy to perform without damaging the thin peritoneal membrane.

On an investigator-developed five-item questionnaire (1–10 Likert scale; ease of dissection, colour preservation, tissue lifelikeness, ease of operating, and overall satisfaction), the F4L cadaver stored three months after fixation achieved the highest mean score (9.34/10), closely followed by the fresh-frozen cadaver (9.20/10), and clearly above the F4L cadaver used immediately after fixation (7.47/10). The F4L-immersion cadaver stored three months after fixation was therefore considered most suitable for future training and protocol refinement. Per-item results are reported in the Supplementary Methods.

## Discussion

This two-phase cadaveric study demonstrates that the anatomical exposure required for da Vinci Xi-assisted multivisceral compartmental resection of right- and left-sided RPS can be achieved in a tumour-free model, with access to all anatomical domains defined in the six-stage open techniques. By explicitly mapping the robotic procedure to these stages, the study moves beyond isolated case reports of retroperitoneal tumour resection and addresses whether a robotic approach can support the full oncologic extent of modern RPS surgery — to our knowledge for the first time [19–23]. The ability of both surgeons to reach all predefined landmarks indicates that these anatomical domains are accessible robotically in a tumour-free model. Whether oncological completeness can be achieved in the presence of a tumour is a separate question that the present design cannot answer. Furthermore, if adequate working space exists, whether the tumour can be mobilized while respecting oncological principles, the posterior planes remain exposable, and if multivisceral resection can be completed en bloc without compromising margins all remain unanswered by these data.

It bears emphasis that the primary objective of RPS surgery is radical oncological resection, not technical accomplishment. A robotic approach warrants consideration only if it delivers the same oncological quality as open surgery, and technical feasibility alone is not a justification for clinical adoption. Any future clinical application must therefore be confined to highly selected patients treated in expert sarcoma centres within multidisciplinary teams, and undertaken only where the robotic approach does not jeopardize the principles of compartmental surgery or the achievement of adequate margins.

Taking into account the currently accepted histology-tailored approach to soft-tissue sarcoma [24], we deliberately tested the maximum possible extent of resection — compartmental multivisceral resection — in the confirmatory phase. Large collaborative analyses have shown that patterns of failure and the prognostic impact of margin status vary substantially by histologic subtype [5, 6]. For well- and dedifferentiated liposarcoma, extended multivisceral resection with ipsilateral organ and fat clearance has been associated with improved disease-specific and overall survival; for leiomyosarcoma, solitary fibrous tumour, malignant peripheral nerve sheath tumour, and undifferentiated pleomorphic sarcoma, negative-margin surgery with selective organ resection based on invasion appears more appropriate. The extent of resection can therefore be tailored to tumour location and histologic subtype — which may make minimally invasive approaches more readily achievable in selected indications. Our aim here, however, was to test the maximum extent, since demonstrating feasibility at this extreme bounds the technique’s applicability.

The side-specific port templates identified here — suprasymphysial linear ports for right-sided resections and an oblique xiphoid-to-right-lower-quadrant line for left-sided resections — are conceptually grounded in established colorectal robotic setups but refocused on the spatial demands of RPS compartmental surgery. They offer a consistent starting point for programmatic implementation and may reduce the trial-and-error phase that accompanies new robotic indications, although reproducibility across teams and institutions remains to be demonstrated. Integration into structured training pathways, including cadaveric and simulator-based curricula, could accelerate the safe learning curve for surgeons transitioning from open to robotic approaches. The F4L-fixed cadavers stored for three months after fixation appear particularly suitable for robotic dissection courses, based on the tactile and dissection properties observed here.

From an oncologic perspective, any move toward robotic RPS surgery must be reconciled with the cautionary stance articulated by sarcoma experts regarding MIS in this setting [4]. Analyses of MIS versus open RPS resection have shown shorter hospital stays without clear short-term survival detriment, but are limited by tumour size, selection bias, and lack of detailed margin and recurrence data [3, 20]. Contemporary single-centre experiences in da Vinci-assisted retroperitoneal tumour surgery report favourable perioperative outcomes and low short-term recurrence, but typically involve smaller tumours (≤ 6–7 cm), benign or low-grade lesions, and infrequent multivisceral resections. By contrast, primary RPS in current series often present with median diameters around 20 cm [5], require extensive organ sacrifice, and carry substantial local recurrence risk even after maximal open surgery.

Our findings should therefore not be interpreted as justification for routine or widespread adoption of robotic surgery for all primary RPS, but rather as a foundational technical proof of concept. In a live setting, a bulky mass will alter exposure, limit working space, and may modify the practicality of approaching key planes beneath the tumour. Even with the robot’s three working trocars plus an auxiliary trocar, mobilizing and holding a large tumour together with colon, mesocolon and kidney such that complete dissection is possible is likely to be challenging, and the feasibility of resecting larger tumours remains to be proven. Specimen extraction will almost always necessitate a sizeable incision, attenuating but not eliminating the classical benefits of MIS. Nonetheless, given that fully robotic donor hepatectomy and robotic liver graft implantation — unthinkable a decade ago — are now performed [25], robotic assistance may offer meaningful advantages in selected RPS scenarios: early-stage or smaller-volume RPS in histology-tailored approaches (e.g., leiomyosarcoma or solitary fibrous tumour, which do not require compartmental resection), and recurrent tumours in which specific vascular or neural structures should be preserved. The high incidence of secondary complications after extensive midline laparotomy — particularly incisional hernias — and the option of replacing it with smaller transverse extraction incisions (e.g., Pfannenstiel) provide further arguments for pursuing minimally invasive approaches in selected cases, given that robotic surgery offers additional advantages over conventional laparoscopy.

### Limitations

The principal limitation of this study is the absence of a tumour; the second is its scale. The confirmatory six-stage dissection was performed once on each side in a single cadaver, by two surgeons from a single centre; no tumour was present, and live conditions (vascular distension, tissue turgor, bleeding, inflammatory response) could not be simulated. The anatomical reach rating is a novel pragmatic instrument developed for this study and has not undergone formal validation. The System Usability Scale was completed as a single joint consensus rating rather than individually by each surgeon. This deviates from the design of the SUS as an individual, user-reported instrument, eliminates inter-user variability, and may have influenced the resulting value; the score of 72.5/100 should therefore be read as a single team-level indication of platform usability in this task rather than as a user-averaged result. No conclusions can be drawn about blood loss, operative time, postoperative outcomes, or long-term oncologic results, and in vivo feasibility for bulky tumours was not addressed. Specifically, this study does not demonstrate oncological equivalence to open surgery, margin adequacy, specimen integrity, en bloc resection capability, or feasibility in the presence of a large retroperitoneal mass. These constraints are appropriate for IDEAL-D Stage 0 but mandate caution in extrapolating findings beyond technical feasibility, and the terms used throughout should be understood in that restricted sense.

### Next steps

Within an IDEAL-aligned development pathway, the next phases are: (i) a cadaveric study incorporating tumour phantoms of varying sizes and locations, designed to reproduce both the volume and mechanical behaviour of a large RPS, currently being planned with our Institute of Anatomy. This phase will test whether the port templates defined here remain valid in the presence of a retroperitoneal mass and will apply objective robotic performance assessment instruments such as GEARS; (ii) prospective documentation of carefully selected clinical cases (smaller tumours, favourable anatomy, multidisciplinary consensus), with detailed reporting of margin status, complications, and early recurrence; and (iii) collaborative work within sarcoma networks to harmonize indications, technical standards, and outcome reporting, analogous to existing consensus processes for open RPS management.

## Conclusion

This study demonstrates that a robotic platform can reproduce the anatomical exposure required for the six-stage compartmental resection concept in a cadaveric model, using consistent side-specific port templates derived from iterative configuration testing. Further investigation is required to determine feasibility, safety, and oncological adequacy in tumour-bearing and clinical settings. These findings cannot yet be extrapolated to the large RPS encountered in clinical practice, where tumour bulk remains the principal obstacle, and technical feasibility alone does not justify clinical adoption.

## Supplementary Information

Below is the link to the electronic supplementary material.


Supplementary Material 1


## Data Availability

All anonymised data generated and analysed during the current study — the structured reach ratings, safety log, SUS responses, and tissue handling questionnaire — are included in this published article and its Supplementary Methods. Further detail is available from the corresponding author upon reasonable request.
