# Supplementary material for: Robotic-assisted compartmental dissection for retroperitoneal sarcoma: a feasibility study on cadavers with the da Vinci Xi system
Source: J Robot Surg. 2026 Aug 29;20(1):906. doi: 10.1007/s11701-026-03869-6 (PMC13526118; doi:10.1007/s11701-026-03869-6)
Supplement: Supplementary file 1 [file 11701_2026_3869_MOESM1_ESM.docx]

**Supplementary Methods**

*Objective Assessment Framework for Cadaveric Robotic Feasibility Study of Compartmental Resection for Retroperitoneal Sarcoma*

# 1. Framework and rationale

This supplement specifies the structured assessment framework used to evaluate the feasibility of robotic compartmental resection for right- and left-sided retroperitoneal sarcoma (RPS) in human cadavers. The framework was designed pragmatically because no single previously validated instrument captures all relevant dimensions of a preclinical cadaveric robotic feasibility study. A layered combination of (i) a graded anatomical reach rating, (ii) a validated usability instrument, and (iii) a structured safety log was therefore used.

**Study classification.** The study corresponds to *IDEAL-D Stage 0* (preclinical device evaluation prior to first-in-human use) within the IDEAL framework for surgical innovation (Hirst et al., Ann Surg 2019; McCulloch et al., Lancet 2009). Reporting follows the *CACTUS guidelines* for cadaveric training and surgical studies (Mantica et al., Int J Surg 2022), and methodological quality was self-appraised against the *QUACS scale* (Wilke et al., J Anat 2015).

# 2. Two-phase study design and assessment allocation

**Phase 1 — Configuration optimization.** Four trocar configurations (oblique with xiphoid to right lower quadrant, oblique with xiphoid to left lower quadrant, vertical paramedian, horizontal suprasymphysial) were trialled iteratively for right- and left-sided access on Cadaver No. 1 and Cadaver No. 2. Dissections in this phase were exploratory and partial; their purpose was to compare port arrangements, not to complete the six-stage protocols. Both surgeons assessed each configuration jointly for ergonomics, arm conflict, external collision, and qualitative reach toward the principal anatomical targets, and selected the final right-sided (suprasymphysial linear) and left-sided (xiphoid-to-right-lower-quadrant oblique) templates by consensus.

**Phase 2 — Confirmatory six-stage feasibility test.** With the optimized port templates in place, the full six-stage compartmental resection protocols of Radaelli et al. (2023; right) and Baia et al. (2025; left) were executed on Cadaver No. 3 — once for the right side and once for the left side (n = 1 confirmatory dissection per side). All structured ratings (anatomical reach rating, safety log, SUS) were applied in this phase.

### Table S1 *Phase and assessment allocation per cadaver.*

| **Cadaver** | **Preservation** | **Phase** | **Purpose** | **Structured assessments applied** |
| --- | --- | --- | --- | --- |
| **No. 1** | Fresh-frozen, thawed before use | Phase 1 | Configuration optimization (right & left port templates) | Tissue-handling questionnaire; qualitative configuration assessment |
| **No. 2** | F4L-immersion, used immediately after fixation | Phase 1 | Configuration optimization (right & left port templates) | Tissue-handling questionnaire; qualitative configuration assessment |
| **No. 3** | F4L-immersion, stored 3 months after fixation | Phase 2 | Confirmatory six-stage dissection: right (n = 1) and left (n = 1) | Anatomical reach rating; safety surrogate log; SUS; tissue-handling questionnaire |

*F4L = Fix for Life (immersion fixation fluid; Fix for Life BV, Leiden, Netherlands).*

**Implications for statistical reporting.** The confirmatory N is small by design (one cadaver, one dissection per side). All structured ratings are therefore reported descriptively (per-landmark scores, per-stage medians) rather than inferentially. Inter-rater agreement for the two-surgeon anatomical reach rating is reported as the proportion of exact agreement and as weighted Cohen's κ per side, acknowledging that the small N constrains the precision of these estimates. The framework is appropriate for IDEAL-D Stage 0 (preclinical proof of concept) and is not intended for hypothesis testing.

# 3. Anatomical reach rating (5-point Likert)

**Status of the instrument.** The anatomical reach rating used here is a *novel pragmatic 5-point Likert instrument developed for this study*. It has not undergone formal psychometric validation. A descriptive label rather than a proprietary acronym is used deliberately, to avoid implying validation that does not exist. No previously validated tool dedicated to graded anatomical reach assessment in robotic cadaveric studies was identified during framework design; the present pragmatic approach with explicit anchors is consistent with current precedent for Stage-0 preclinical evaluations.

**Why a graded rather than binary scale.** Because the confirmatory dissection succeeded for all landmarks, a binary completed / not-completed classification would have yielded uninformative all-completed data. The 5-point graded scale captures the variation that is actually present (full unrestricted reach vs. reach only after redocking vs. reach requiring significant compensatory manoeuvres) and preserves the binary information: a score of 1 corresponds to no useful reach (effectively not completed), and a score of ≥ 2 corresponds to some degree of completed access.

**Administration.** Applied to the Phase 2 confirmatory dissections only (Cadaver No. 3, right and left). Each landmark was rated independently by both surgeons after completion of the relevant stage. Where a landmark received a score < 5, the limiting factor was recorded from a closed list: R = reach, A = arm conflict / external collision, V = visualization, T = tissue limitation, O = other (free text).

## 3.1 Rating rubric

| **Score** | **Anchor** | **Description** |
| --- | --- | --- |
| **5** | **Full reach** | Full access and unrestricted dissection achieved with optimal instrument angulation; no port or docking adjustment required; no arm conflict. |
| **4** | **Good reach** | Access achieved and dissection step completed; minor angulation/exposure compromise or minor external collision tolerated without changing ports or docking. |
| **3** | **Reach with adjustment** | Access achieved only after port repositioning, redocking, target re-anatomy, or significant boom/arm adjustment. |
| **2** | **Partial reach** | Partial access only; landmark visualized and partly dissected, but some aspects of the predefined dissection step could not be completed robotically. |
| **1** | **No useful reach** | No useful access; landmark not reachable in the current configuration; step would have required conversion in a live setting. |

*Limiting-factor codes (recorded for any landmark scoring < 5): R = reach; A = arm conflict / external collision; V = visualization; T = tissue limitation; O = other (free text).*

## 3.2 Right-sided RPS — anatomical reach rating sheet (Phase 2, Cadaver No. 3)

### Table S2 *Right-sided per-landmark reach scores, rated independently by both surgeons.*

| **Stage** | **Landmark** | **S1** | **S2** | **Limiting code** | **Notes / mechanism** |
| --- | --- | --- | --- | --- | --- |
| **Stage 1** | Adequate exposure of the whole abdominal cavity — diagnostic survey of peritoneal surfaces | **3** | **5** | O | Performed laparoscopically; limited circumferential visibility after docking |
| **Stage 2** | Division of the gastrocolic ligament | **5** | **5** | — | — |
|  | Mobilization of the transverse colon | **5** | **5** | — | — |
|  | Visualization of right branch of middle colic vessels | **5** | **5** | V | Missing diaphanoscopy in MIS |
|  | Identification and ligation of ileocolic vessels | **5** | **5** | — | — |
|  | Exposure of SMV and SMA | **4** | **5** | A | Minor arm conflict (Surgeon 1) |
| **Stage 3** | Full Kocher manoeuvre with duodenal mobilization | **5** | **5** | — | — |
|  | Exposure of the pancreatic head | **5** | **5** | — | — |
|  | Safe dissection along the duodenum | **5** | **5** | — | — |
|  | Posterior dissection toward the vena cava | **4** | **4** | A | Minor arm conflict on caval rotation |
| **Stage 4** | Dissection along the inferior vena cava | **5** | **5** | — | — |
|  | Central ligation of right gonadal vessels | **5** | **5** | — | — |
|  | Identification and ligation of the right renal artery | **5** | **5** | — | — |
|  | Identification and ligation of the right renal vein | **5** | **5** | — | — |
| **Stage 5** | Lateral peritonectomy | **4** | **4** | A | Steep instrument angle into pelvis |
|  | Exposure of iliac vessels | **4** | **4** | A | Steep instrument angle into pelvis |
|  | Identification of the right ureter | **4** | **4** | A | Steep instrument angle into pelvis |
|  | Peripheral ligation of gonadal vessels | **4** | **4** | A | — |
|  | Identification and preservation of obturator nerve | **4** | **4** | A | — |
|  | Identification and preservation of femoral nerve | **4** | **4** | A | — |
|  | Partial distal division of the psoas muscle | **4** | **4** | A | — |
| **Stage 6** | Dissection along the spine | **5** | **5** | — | — |
|  | Retrocaval dissection | **5** | **5** | — | — |
|  | Partial resection of psoas muscle | **5** | **5** | — | — |
|  | Exposure of right adrenal vein / right adrenalectomy | **5** | **5** | — | — |
|  | Mobilization of right diaphragmatic fold and crus | **4** | **4** | R | Cranial reach to crus |

*S1 = Surgeon 1; S2 = Surgeon 2. Shaded score cells mark the two landmarks where the surgeons' scores differed (inter-rater agreement 24/26 = 92.3%; see section 3.4). Code: R = reach; A = arm conflict; V = visualization; T = tissue limitation; O = other. SMA superior mesenteric artery, SMV superior mesenteric vein.*

***Per-stage summary, right side (pooled across both surgeons):***

Stage 1 — median 4 (range 3–5; 1 landmark, 2 ratings); Stage 2 — median 5 (range 4–5; 5 landmarks, 10 ratings); Stage 3 — median 5 (range 4–5; 4 landmarks, 8 ratings); Stage 4 — median 5 (4 landmarks, 8 ratings); Stage 5 — median 4 (7 landmarks, 14 ratings); Stage 6 — median 5 (range 4–5; 5 landmarks, 10 ratings). Overall: median 5 (range 3–5; 26 landmarks, 52 ratings; score distribution 5/4/3 = 32/19/1).

## 3.3 Left-sided RPS — anatomical reach rating sheet (Phase 2, Cadaver No. 3)

### Table S3 *Left-sided per-landmark reach scores, rated independently by both surgeons.*

| **Stage** | **Landmark** | **S1** | **S2** | **Limiting code** | **Notes / mechanism** |
| --- | --- | --- | --- | --- | --- |
| **Stage 1** | Adequate exposure of the whole abdominal cavity | **3** | **5** | O | Performed laparoscopically; limited circumferential visibility after docking |
|  | Identification of IMV at the duodenojejunal junction | **5** | **5** | — | — |
| **Stage 2** | Division of the transverse colon | **5** | **5** | — | — |
|  | Assessment of the pancreas and spleen | **5** | **5** | — | — |
|  | Division of the gastrocolic ligament | **5** | **5** | — | — |
|  | Ligation of the inferior mesenteric vein (IMV) | **5** | **5** | — | — |
| **Stage 3** | Liberation of the pancreatic body/tail | **4** | **4** | R | Cranial reach |
|  | (If indicated) Resection of pancreatic tail | **4** | **4** | R | Cranial reach |
|  | Division of the splenorenal ligament | **4** | **4** | R | Cranial reach |
|  | Release of diaphragmatic attachments | **4** | **4** | R | Cranial reach |
|  | Exposure of the left retroperitoneum | **5** | **5** | — | — |
| **Stage 4** | Aortic dissection | **5** | **5** | — | — |
|  | Ligation of the left renal artery | **5** | **5** | — | — |
|  | Ligation of the left renal vein | **5** | **5** | — | — |
|  | Ligation of the inferior mesenteric artery (IMA) | **5** | **5** | — | — |
| **Stage 5** | Division of the rectum | **3** | **4** | R | Planned redocking from splenic flexure onto the sigmoid |
|  | Division of the distal psoas | **5** | **5** | — | — |
|  | Peritonectomy up to the costodiaphragmatic fold | **4** | **4** | R | Cranial reach |
|  | Ligation of gonadal vessels | **5** | **5** | — | — |
|  | Identification and ligation of the left ureter | **5** | **5** | — | — |
|  | Dissection of iliac vessels | **4** | **5** | A | Arm conflict in pelvis (Surgeon 1) |
|  | Isolation of the femoral nerve | **4** | **4** | A | Arm conflict in pelvis |
| **Stage 6** | Dissection of the psoas muscle | **5** | **5** | — | — |
|  | Liberation of the costodiaphragmatic fold | **4** | **4** | R | Cranial reach |

*S1 = Surgeon 1; S2 = Surgeon 2. Shaded score cells mark the three landmarks where the surgeons' scores differed (inter-rater agreement 21/24 = 87.5%; see section 3.4). Code: R = reach; A = arm conflict; V = visualization; T = tissue limitation; O = other. IMV inferior mesenteric vein, IMA inferior mesenteric artery.*

***Per-stage summary, left side (pooled across both surgeons):***

Stage 1 — median 5 (range 3–5; 2 landmarks, 4 ratings); Stage 2 — median 5 (4 landmarks, 8 ratings); Stage 3 — median 4 (range 4–5; 5 landmarks, 10 ratings); Stage 4 — median 5 (4 landmarks, 8 ratings); Stage 5 — median 4.5 (range 3–5; 7 landmarks, 14 ratings); Stage 6 — median 4.5 (range 4–5; 2 landmarks, 4 ratings). Overall: median 5 (range 3–5; 24 landmarks, 48 ratings; score distribution 5/4/3 = 30/16/2).

## 3.4 Inter-rater agreement

Both surgeons rated all 50 landmarks (26 right, 24 left) independently. Agreement was summarized as the proportion of exact agreement and as Cohen's κ. Because the rating is ordinal, κ was computed with both linear and quadratic distance weights; the unweighted κ is reported for completeness. Confidence intervals were obtained by non-parametric bootstrap (10 000 landmark-level resamples with replacement; percentile method).

### Table S4 *Inter-rater agreement between the two surgeons for the anatomical reach rating.*

| **Side** | **Exact agreement** | **Unweighted κ** | **Linear-weighted κ** | **Quadratic-weighted κ (95% CI)** |
| --- | --- | --- | --- | --- |
| **Right (n = 26)** | 24/26 (92.3%) | 0.84 | 0.78 | 0.66 (0.28–1.00) |
| **Left (n = 24)** | 21/24 (87.5%) | 0.75 | 0.70 | 0.63 (0.28–1.00) |
| **Pooled (n = 50)** | **45/50 (90.0%)** | **0.80** | **0.74** | **0.64 (0.38–0.93)** |

*n = number of landmarks rated by each surgeon. Linear-weighted κ 95% CIs (bootstrap): right 0.48–1.00, left 0.41–1.00, pooled 0.54–0.92. κ computed over the score categories observed (3, 4, 5); categories 1 and 2 did not occur and do not affect the estimate.*

**Disagreements.** The five landmarks where the surgeons differed (shaded in Tables S2 and S3) were: right Stage 1 access and exploration (S1 = 3, S2 = 5) and right Stage 2 exposure of SMV/SMA (S1 = 4, S2 = 5); left Stage 1 access and exploration (S1 = 3, S2 = 5), left Stage 5 division of the rectum (S1 = 3, S2 = 4), and left Stage 5 dissection of the iliac vessels (S1 = 4, S2 = 5). Four of the five were single-point disagreements. The only two-point disagreement was the Stage 1 access-and-exploration landmark on each side, where the surgeons differed in how they scored circumferential visibility after robotic docking — a step performed laparoscopically in both dissections.

**Interpretation.** By the Landis and Koch (1977) benchmarks, exact agreement of 87.5–92.3% and κ values of 0.63–0.84 indicate substantial-to-almost-perfect agreement. The quadratic-weighted κ is lower than the unweighted and linear-weighted values because quadratic weighting penalizes the two-point Stage 1 disagreement disproportionately while the scores are otherwise highly concentrated (97% of all ratings are 4 or 5). With concentrated marginal distributions, κ is known to be conservative relative to the observed agreement (the so-called "kappa paradox"); the proportion of exact agreement is therefore reported alongside κ as the more directly interpretable statistic. The confidence intervals are wide because the confirmatory dissection comprised a single procedure per side (n = 1); the agreement statistics are descriptive and are not intended to support inferential claims, consistent with the IDEAL-D Stage 0 design.

# 4. System Usability Scale (SUS) — adapted

**Source.** Brooke J. SUS: a 'quick and dirty' usability scale. In: Jordan PW, Thomas B, Weerdmeester BA, McClelland AL, eds. *Usability Evaluation in Industry*. London: Taylor & Francis; 1996. p. 189–94. Interpretation thresholds based on Bangor A, Kortum P, Miller J. An empirical evaluation of the System Usability Scale. *Int J Hum-Comput Interact*. 2008;24(6):574–94.

**Adaptation.** The original SUS refers generically to "the system". For this study, every occurrence is replaced consistently by "the Da Vinci Xi for robotic compartmental dissection of retroperitoneal sarcoma". This wording substitution is the only change; item count, polarity alternation, and scoring procedure are preserved unchanged. Single-term substitution preserves the established psychometric properties of the SUS (Bangor et al. 2008; Lewis JR, Int J Hum-Comput Interact 2018;34(7):577–90).

**Administration.** The SUS was completed once, as a joint consensus rating by both surgeons, following the Phase 2 confirmatory dissections, capturing the cumulative experience with the system across both phases. The agreed responses are reproduced in Table S5, incorporating the post-hoc correction to item 6 described below.

**Scoring.** Each item is rated 1 (Strongly disagree) to 5 (Strongly agree). For odd-numbered items, contribution = (response – 1). For even-numbered items, contribution = (5 – response). Sum the contributions (0–40) and multiply by 2.5 to obtain the SUS score (0–100). Conventional benchmarks: < 50 = unacceptable; 50–67 = marginal; 68 = average; ≥ 80.3 = excellent (Bangor et al. 2008).

## 4.1 SUS items (adapted) and recorded responses

### Table S5 *Adapted SUS items and recorded responses.*

| **#** | **Statement** | **1** | **2** | **3** | **4** | **5** |
| --- | --- | --- | --- | --- | --- | --- |
| **1** | I think that I would like to use the Da Vinci Xi for robotic compartmental dissection of retroperitoneal sarcoma frequently. | □ | □ | **X** | □ | □ |
| **2** | I found the Da Vinci Xi for robotic compartmental dissection of retroperitoneal sarcoma unnecessarily complex. | □ | **X** | □ | □ | □ |
| **3** | I thought the Da Vinci Xi for robotic compartmental dissection of retroperitoneal sarcoma was easy to use. | □ | □ | □ | **X** | □ |
| **4** | I think that I would need the support of a technical person to be able to use the Da Vinci Xi for robotic compartmental dissection of retroperitoneal sarcoma. | **X** | □ | □ | □ | □ |
| **5** | I found the various functions in the Da Vinci Xi for robotic compartmental dissection of retroperitoneal sarcoma were well integrated. | □ | □ | □ | □ | **X** |
| **6** | I thought there was too much inconsistency in the Da Vinci Xi for robotic compartmental dissection of retroperitoneal sarcoma. | **X** | □ | □ | □ | □ |
| **7** | I would imagine that most people would learn to use the Da Vinci Xi for robotic compartmental dissection of retroperitoneal sarcoma very quickly. | □ | □ | □ | **X** | □ |
| **8** | I found the Da Vinci Xi for robotic compartmental dissection of retroperitoneal sarcoma very cumbersome to use. | □ | **X** | □ | □ | □ |
| **9** | I felt very confident using the Da Vinci Xi for robotic compartmental dissection of retroperitoneal sarcoma. | □ | □ | **X** | □ | □ |
| **10** | I needed to learn a lot of things before I could get going with the Da Vinci Xi for robotic compartmental dissection of retroperitoneal sarcoma. | □ | □ | □ | **X** | □ |

*Scale anchors: 1 = Strongly disagree; 2 = Disagree; 3 = Neutral; 4 = Agree; 5 = Strongly agree. X = response agreed by consensus of both surgeons. Item 6 was corrected from 3 to 1 after review (the platform was, on reflection, judged not to show meaningful inconsistency); all other items are unchanged. Item-level computation with the corrected value: odd items (response – 1) and even items (5 – response) give (3–1) + (5–2) + (4–1) + (5–1) + (5–1) + (5–1) + (4–1) + (5–2) + (3–1) + (5–4) = 2 + 3 + 3 + 4 + 4 + 4 + 3 + 3 + 2 + 1 = 29. SUS score = 29 × 2.5 = 72.5/100 — above the average benchmark of 68 and within the acceptable range per Bangor et al. (2008). Before the item-6 correction the sum was 27 and the score 67.5; the correction therefore raised the score by 5.0 points.*

# 5. Safety surrogate log

One row per critical structure, completed during the Phase 2 confirmatory dissections (Cadaver No. 3). Recorded by both surgeons in consensus at the end of each dissection.

| **Critical structure** | **Unintended contact?** | **Visible damage?** | **Mechanism (free text)** |
| --- | --- | --- | --- |
| Aorta | **Y** | **N** |  |
| Inferior vena cava | **Y** | **Y** | *Impression of the grasper (no transection or perforation)* |
| Right renal artery / vein | **Y** | **N** |  |
| Left renal artery / vein | **N** | **N** |  |
| Superior mesenteric artery | **N** | **N** |  |
| Superior mesenteric vein | **N** | **N** |  |
| Inferior mesenteric artery | **N** | **N** |  |
| Inferior mesenteric vein | **Y** | **N** |  |
| Iliac artery and vein (R / L) | **N** | **N** |  |
| Right ureter | **N** | **N** |  |
| Left ureter | **Y** | **N** |  |
| Femoral nerve (R / L) | **N** | **N** |  |
| Obturator nerve (R / L) | **N** | **N** |  |
| Duodenum / pancreatic head | **Y** | **N** |  |
| Spleen / pancreatic tail | **N** | **N** |  |
| Bowel (any segment) | **Y** | **Y** | *Impression of the grasper (no transection or perforation)* |

*Y = yes; N = no. Audited n = 16 critical sites. Unintended contact: 7/16 (aorta, IVC, right renal vessels, IMV, left ureter, duodenum/pancreatic head, bowel) — all expected during compartmental dissection. Visible damage: 2/16 (IVC and bowel — grasper impressions without transection or perforation); the remaining 14 sites showed no visible damage.*

# 6. Cadaver tissue handling

Cadaver tissue handling and suitability were assessed with a single five-item questionnaire scored on a 1–10 Likert scale (1 = completely disagree, 10 = completely agree). The instrument was investigator-developed and was oriented on the domains of the McMaster Embalming Scale (Wang et al., Clin Anat 2023), but it is not the validated McMaster Embalming Scale itself and was not administered as such; a descriptive label is therefore used. The five items were: (1) ease of preparation/operating, (2) colour preservation, (3) tissue structure comparable to that of a living person, (4) ease of performing the operation, and (5) overall satisfaction with work on the body. Each item was rated for every cadaver / preservation method by all participating raters.

### Table S6 *Per-item mean responses by preservation method (five-item tissue-handling questionnaire, 1–10 scale).*

| **Item** | **Fresh-frozen** | **F4L immediate** | **F4L body bag 3 mo** |
| --- | --- | --- | --- |
| 1. The tissue is easy to prepare / operate on. | 9.33 | 8.00 | 9.00 |
| 2. The tissue retained its colour well. | 8.67 | 7.33 | 9.29 |
| 3. Tissue structure comparable to that of a living person. | 8.67 | 7.00 | 8.71 |
| 4. The operation can be performed easily on this body. | 9.67 | 7.67 | 9.86 |
| 5. Overall satisfaction with work on this body. | 9.67 | 7.33 | 9.86 |
| **Mean of means** | **9.20** | **7.47** | **9.34** |

*Number of respondents per method: Fresh-frozen n = 3; F4L used immediately after fixation n = 3; F4L stored 3 months after fixation n = 7. Per-item values are arithmetic means of individual ratings.*

# 7. References cited in this supplement

1. Bangor A, Kortum P, Miller J. An empirical evaluation of the System Usability Scale. Int J Hum-Comput Interact. 2008;24(6):574–94. https://doi.org/10.1080/10447310802205776
2. Baia M, Dossa F, Radaelli S, Callegaro D, Colombo C, Borghi A, et al. Six Surgical Stages in the Resection of Primary Left Retroperitoneal Liposarcoma: A Standardized Comprehensive
3. Brooke J. SUS: a 'quick and dirty' usability scale. In: Jordan PW, Thomas B, Weerdmeester BA, McClelland AL, editors. Usability Evaluation in Industry. London: Taylor & Francis; 1996. p. 189–94.
4. Hirst A, Philippou Y, Blazeby J, Campbell B, Campbell M, Feinberg J, et al. No Surgical Innovation Without Evaluation: Evolution and Further Development of the IDEAL Framework and Recommendations. Ann Surg. 2019;269(2):211–20. https://doi.org/10.1097/SLA.0000000000002794
5. Landis JR, Koch GG. The measurement of observer agreement for categorical data. Biometrics. 1977;33(1):159–74. PMID: 843571.
6. Lewis JR. The System Usability Scale: Past, Present, and Future. Int J Hum-Comput Interact. 2018;34(7):577–90.
7. Mantica G, Leonardi R, Diaz R, Malinaric R, Parodi S, Tappero S, et al. Reporting ChAracteristics of cadaver training and sUrgical studies: The CACTUS guidelines. Int J Surg. 2022;101:106619. https://doi.org/10.1016/j.ijsu.2022.106619
8. McCulloch P, Altman DG, Campbell WB, Flum DR, Glasziou P, Marshall JC, et al. No surgical innovation without evaluation: the IDEAL recommendations. Lancet. 2009;374(9695):1105–12. https://doi.org/10.1016/S0140-6736(09)61116-8
9. Radaelli S, Baia M, Drohan A, Morosi C, Sangalli C, Colombo C, et al. Six Surgical Stages in the Resection of Primary Right Retroperitoneal Liposarcoma: A Standardized Comprehensive Approach. Ann Surg Oncol. 2023;30(11):6896–7. https://doi.org/10.1245/s10434-023-13660-8
10. Wang A, de Sa D, Darie S, Zhang B, Rockarts J, Palombella A, et al. Development of the McMaster Embalming Scale (MES) to assess embalming solutions for surgical skills training. Clin Anat. 2023;36(5):754–63. https://doi.org/10.1002/ca.24037
11. Wilke J, Krause F, Niederer D, Engeroff T, Nürnberger F, Vogt L, Banzer W. Appraising the methodological quality of cadaveric studies: validation of the QUACS scale. J Anat. 2015;226(5):440–6. https://doi.org/10.1111/joa.12292
